# Effects of Hormone Therapy on Brain Volumes Changes of Postmenopausal Women Revealed by Optimally-Discriminative Voxel-Based Morphometry

**DOI:** 10.1371/journal.pone.0150834

**Published:** 2016-03-14

**Authors:** Tianhao Zhang, Ramon Casanova, Susan M. Resnick, JoAnn E. Manson, Laura D. Baker, Claudia B. Padual, Lewis H. Kuller, R. Nick Bryan, Mark A. Espeland, Christos Davatzikos

**Affiliations:** 1 Center for Biomedical Image Computing and Analytics, Department of Radiology, University of Pennsylvania, Philadelphia, Pennsylvania, United States of America; 2 Department of Biostatistical Sciences, Wake Forest School of Medicine, Winston-Salem, North Carolina, United States of America; 3 Laboratory of Behavioral Neuroscience, National Institute on Aging, Baltimore, Maryland, United States of America; 4 Division of Preventive Medicine, Department of Medicine, Brigham and Women’s Hospital and Harvard Medical School, Boston, Massachusetts, United States of America; 5 Department of Epidemiology, Harvard School of Public Health, Boston, Massachusetts, United States of America; 6 Department of Internal Medicine and Epidemiology, Wake Forest School of Medicine, Winston-Salem, North Carolina, United States of America; 7 Sierra Pacific Mental Illness Research, Education and Clinical Center, VA Palo Alto Health Care System, Palo Alto, California, United States of America; 8 Department of Psychiatry and Behavioral Sciences, Stanford University, Stanford, California, United States of America; 9 Department of Epidemiology, Graduate School of Public Health, University of Pittsburgh, Pittsburgh, Pennsylvania, United States of America; Le Fe Health Research Institute, SPAIN

## Abstract

**Backgrounds:**

The Women's Health Initiative Memory Study Magnetic Resonance Imaging (WHIMS-MRI) provides an opportunity to evaluate how menopausal hormone therapy (HT) affects the structure of older women’s brains. Our earlier work based on region of interest (ROI) analysis demonstrated potential structural changes underlying adverse effects of HT on cognition. However, the ROI-based analysis is limited in statistical power and precision, and cannot provide fine-grained mapping of whole-brain changes.

**Methods:**

We aimed to identify local structural differences between HT and placebo groups from WHIMS-MRI in a whole-brain refined level, by using a novel method, named Optimally-Discriminative Voxel-Based Analysis (ODVBA). ODVBA is a recently proposed imaging pattern analysis approach for group comparisons utilizing a spatially adaptive analysis scheme to accurately locate areas of group differences, thereby providing superior sensitivity and specificity to detect the structural brain changes over conventional methods.

**Results:**

Women assigned to HT treatments had significant Gray Matter (GM) losses compared to the placebo groups in the anterior cingulate and the adjacent medial frontal gyrus, and the orbitofrontal cortex, which persisted after multiple comparison corrections. There were no regions where HT was significantly associated with larger volumes compared to placebo, although a trend of marginal significance was found in the posterior cingulate cortical area. The CEE-Alone and CEE+MPA groups, although compared with different placebo controls, demonstrated similar effects according to the spatial patterns of structural changes.

**Conclusions:**

HT had adverse effects on GM volumes and risk for cognitive impairment and dementia in older women. These findings advanced our understanding of the neurobiological underpinnings of HT effects.

## Introduction

The Women’s Health Initiative Memory Study (WHIMS) provided a unique opportunity for researchers to examine critical questions regarding the effects of hormone therapy (HT) on brain structure of postmenopausal women. Results from the WHIMS study [1, 2, 3, 4] indicated that conjugated equine estrogens, with and without progestin, increase the risk of dementia and have adverse effects on cognition in women aged 65 and over.

Advances in Magnetic Resonance Imaging (MRI) make it possible to non-invasively and sensitively measure pathologic changes in cortical and subcortical brain parenchyma. WHIMS-MRI, as a sub-study of the Women’s Health Initiative (WHI) and WHIMS, was able to provide a comprehensive examination of the effects of hormone therapy on regional brain structure in postmenopausal women. In our earlier work [5], we investigated whether regions of interest (ROI) including total brain, hippocampus, frontal lobe, and others labeled on MRI scans acquired post-trial, show significant differences in volumes for older women who had been assigned to HT compared with those assigned to placebo. The results suggested that women assigned to HT had decreased volumes in specific regions compared with those assigned to placebo, offering a potential mechanism underlying the adverse effects of HT on cognition. However, the ROI-based analysis may lack statistical precision as it does not take into account the complex and anisotropic structural information circumscribed by the brain ROIs. Moreover, the outputs of the ROI analysis can only provide gross information on volumetric structure of particular regions, which cannot meet the current need of fine-grained mapping of brain alterations.

The current study aimed to identify local structural differences between HT and placebo groups from WHIMS-MRI, using a voxel-wise method, which analyzes the whole brain automatically, resulting in a brain map that reflects statistical significance on a refined level. Voxel-Based Morphometry (VBM) [6, 7, 8] is one such technique, commonly used in past years for mapping neuroanatomical differences including those associated with HT [9, 10, 11]. However, the conventional VBM method has technical shortcomings. First, the general linear model (GLM [12]) exploited in VBM has limited statistical power due to its mass-univariate nature that discards complex multivariate relationships in the data [13]. Second, the Gaussian smoothing, typically applied prior to the GLM step in VBM, has been shown to increase the risk of both false positive and false negative results [14, 15, 16] due to its blurring effects on the spatial signals of images.

In this study we used a novel method, termed optimally-discriminative voxel-based analysis (ODVBA), which is a recently proposed imaging pattern analysis approach for group comparisons recently proposed by [17, 18]. ODVBA utilizes a spatially adaptive analysis scheme to accurately locate areas of group differences, and thereby transcends the limitations of the commonly used Gaussian smoothing with a fixed kernel size that precedes GLM, translating to superior sensitivity and specificity to detect the structural brain changes. The performance of ODVBA has been extensively validated in both the simulated data in which the ground truth on the simulated abnormalities is known [17] and the real data from clinical studies in Alzheimer's Disease [17, 18], schizophrenia [18, 19], ADHD [20], imaging effects of diabetes [21], among others. To our knowledge, the present study is the first voxel-based morphometric study to investigate the structural brain changes associated with hormone therapy in the WHIMS project.

## Materials and Methods

### Participants

As an ancillary study of WHI (registered at ClinicalTrials.gov with ID# NCT00000611), WHIMS was conducted to investigate the effects of hormone therapy on risk of dementia and changes in cognitive function in women aged 65 or older. Data are available from Women's Health Initiative (www.whi.org), once a signed data use agreement is in place. For more details refer to the associated Data Availability statement. The subjects were randomized into the parallel placebo-controlled randomized clinical trials of 0.625 mg/d conjugated equine estrogens (CEE) therapy alone (CEE-Alone) and in combination with 2.5 mg/d medroxyprogesterone acetate (CEE+MPA). The study protocols and consent forms were approved by National Institutes of Health and Institutional Review Boards of all 40 participating clinical centers (https://www.nhlbi.nih.gov/whi/ccenterlist.htm). Written informed consent was obtained from each participant. Participant records/information was anonymized and de-identified prior to analysis. The details of this study were previously published [1, 2, 22].

The WHIMS-MRI trial, conducted at 14 of 39 WHIMS sites [5, 23], was designed to determine whether ischemic lesion volumes and volumes of brain parenchym differed between women previously randomized to CEE-Alone and CEE+MPA and their respective placebo groups. For the overall with trial, randomization was stratified by site to achieve balance. Among participants included in our analysis, treatment assignments were balanced among clinics (*p* = 0.32). The recruitment started in January 2005 and ended in April 2006, approximately 1.4 years for CEE-Alone and 3.0 years for CEE+MPA after the termination of the WHI trial. Of the total sample of 1424 participants who completed the scanning, 1,365 participants who met the reading criteria were included in this analysis, including 254 active and 256 placebo participants in the CEE-Alone trial, and 420 active and 435 placebo participants in the CEE+MPA trial, making the total 674 HT and 691 placebo participants. The demographic, lifestyle, and clinical characteristics of the women included in this study are listed in Table 1. As demonstrated, there were no marked differences in the related risk factors between the active treatment groups and the associated placebo.

**Table 1:** Demographic, lifestyle, and clinical characteristics of WHIMS-MRI women by treatment assignment.

Variable	WHIMS-MRI: CEE-Alone		WHIMS-MRI: CEE+MPA		Statistics (*p* values, %)		
	CEE-Alone (n = 254)	Placebo (n = 256)	CEE+MPA (n = 420)	Placebo (n = 435)	CEE-Alone vs. Placebo	CEE+MPA vs. Placebo	HT vs. Placebo
**Age**, no. (%)					90.82	87.94	87.33
65–69 y	123(48.43)	128(50.00)	220 (52.38)	225 (51.72)			
70–74 y	93(36.61)	89 (34.77)	147 (35.00)	150 (34.48)			
75 + y	38 (14.96)	39 (15.23)	53 (12.62)	60 (13.79)			
**Ethnicity**					9.63	27.65	97.32
Black/African American	13 (5.16)	16 (6.27)	17 (4.05)	15 (3.46)			
White, non-Hispanic	222 (88.10)	232(90.98)	392 (93.33)	399 (91.94)			
Other	17 (6.75)	7 (2.75)	11 (2.62)	22 (4.61)			
**Education**					13.05	51.59	94.43
<High school	16 (6.30)	8 (3.13)	15 (3.58)	21 (4.85)			
High school /GED	69 (27.17)	69 (26.95)	84 (20.05)	95 (21.94)			
>High school, <4 y college	94 (37.10)	115 (44.92)	174 (41.53)	161 (37.18)			
>4 y college	75 (29.53)	64 (25.00)	146 (34.84)	156 (36.03)			
**Smoking Status**					44.90	63.60	69.82
Never	147 (58.57)	142 (55.69)	248 (59.33)	247 (57.44)			
Former	95 (37.85)	98 (38.43)	152 (36.36)	168 (39.07)			
Current	9 (3.59)	15 (5.88)	18 (4.31)	15 (3.49)			
**Body Mass Index**					77.45	69.06	73.06
<25 kg/m^2^	59 (23.41)	63 (24.71)	134 (31.98)	153 (35.25)			
25–29 kg/m^2^	98 (38.89)	107 (41.96)	157 (37.47)	151 (34.79)			
30–34 kg/m^2^	60 (23.81)	55 (21.57)	89 (21.24)	86 (19.82)			
≥35 kg/m^2^	35 (13.89)	30 (11.76)	39 (9.31)	44 (10.14)			
**Hypertension Status**					38.11	24.42	68.68
None	120 (47.24)	134 (52.34)	227 (54.05)	234 (53.79)			
Current/controlled	51 (20.08)	53 (20.70)	53 (12.62)	45 (10.34)			
Current/uncontrolled	83 (32.68)	69 (26.95)	140 (33.33)	156 (35.86)			
**Prior Cardiovascular Disease**					92.22	20.95	36.59
No	233 (91.73)	233 (91.02)	399 (95.00)	414 (95.17)			
History of stroke	3 (1.18)	4 (1.56)	1 (0.24)	5 (1.15)			
History of other CVD	18 (7.09)	19 (7.42)	20 (4.76)	16 (3.68)			
**Diabetes**					49.72	36.05	22.47
No	238 (93.70)	235 (91.80)	405 (96.43)	414 (95.17)			
Yes	16 (6.30)	21 (8.20)	15 (3.57)	21 (4.83)			
**Prior Hormone Therapy**					59.56	70.42	58.78
No	129 (50.79)	124 (48.44)	328 (78.10)	335 (77.01)			
Yes	125 (49.21)	132 (51.56)	92 (21.90)	100 (22.99)			
**3MS score**					77.10	46.60	38.40
<90	20 (7.94)	19 (7.48)	20 (4.77)	17 (3.96)			
90–94	50 (19.84)	57 (22.44)	63 (15.04)	77 (17.95)			
95–100	182 (74.22)	178 (70.08)	336 (80.19)	335 (78.09)			

### Image Acquisition and Pre-Processing

MRI scans were performed using a standardized protocol which was developed by investigators at the MRI Quality Control (QC) Center in the Department of Radiology of University of Pennsylvania (UPenn). The scanners were standardized by American College of Radiology (ACR) phantom QC protocol as follows. Before each clinical site can enroll subjects into WHIMS study protocol, a set of test scans on the ACR QC phantoms [24] and a volunteer have been be submitted for review and approved by the MRI QC Center in UPenn. The ACR phantom tests have been performed on a quarterly basis. Details on procedures for acquisition and processing were provided previously [5, 25]. Briefly, the scans were obtained with a field of view = 22 cm and a matrix of 256×256. Included were oblique axial spin density/T2-weighted spin echo (TR:3200 ms, TE = 30/120 ms, slice thickness = 3 mm), fluid-attenuated inversion recovery (FLAIR) T2-weighted spin echo (TR = 8000 ms, TI = 2000 ms, TE = 100 ms, slice thickness = 3 mm), and oblique axial three-dimensional T1-weighted gradient echo (flip angle = 30 degrees, TR = 21 ms, TE = 8 ms, slice thickness = 1.5 mm) images from the vertex to the skull base parallel to the anterior commissure–posterior commissure (AC-PC) plane.

The T1-weighted images were preprocessed according to a number of steps including 1) alignment of the brain with the AC-PC plane by manually; 2) removal of extra-cranial material using the BET method [26]; 3) tissue segmentation into gray matter (GM), white matter (WM), and cerebrospinal fluid (CSF), using a method described in [27]; 4) high-dimensional image warping to a standard MNI space [28], through an elastic registration method [29]; 5) applying the deformation field that resulted from the spatial registration to the segmented images, thereby generating mass-preserved volumetric maps (or tissue density maps), named Regional Analysis of Volumes Examined in Normalized Space (RAVENS) maps (Davatzikos et al., 2001); and 6) correction for the effects of i) intracranial volume (ICV), ii) age, iii) clinic site, and iv) time from randomization to scan, using the multiple linear regression model [30].

### Statistical Analysis

Group comparisons were performed via voxel-based statistical analysis of the volumetric measurements by using ODVBA. The ODVBA software is freely available (https://www.cbica.upenn.edu/sbia/software/odvba/) under a BSD-style open source license. ODVBA is a recently proposed imaging pattern analysis framework used to more accurately detect the group differences in brain imaging data. ODVBA starts with the regional multivariate discriminative analyses with a non-negativity constraint, as an optimal anisotropic filtering of images that enhances group differences. Next, the whole brain map of statistic is calculated by tallying the weights of each voxel to all of the neighborhoods in which it belongs. Finally, the statistical significance maps are obtained via nonparametric permutation testing [31]: assuming that the null hypothesis is that there is no difference between two groups, the *p* value of each voxel is calculated by comparing the observed statistic to the random permutation distribution. The current study used 2,000 permutations to derive the significances.

To address the multiple-comparison problem, we implemented cluster-wise Family Wise Error (FWE) correction based on non-parametric permutation tests [31, 32, 33]. The cluster-wise FWE is reported to be more powerful [32] than the voxel-wise False Discovery Rate (FDR) correction [34, 35], because it takes into account the spatial correlations [33] within regional volumes. The resulting maps of significance were partitioned and analyzed according to the Automated Anatomical Labeling (AAL) package [36]. On each anatomical region, we calculated the cluster size, the *t* statistic (based on the means of the tissue density of the detected area per region, as well as in [17]), and the Talairach coordinate of the mass’s center [37].

## Results

### GM Findings

#### HT, CEE-Alone, and CEE+MPA < Placebo

**Fig 1:**
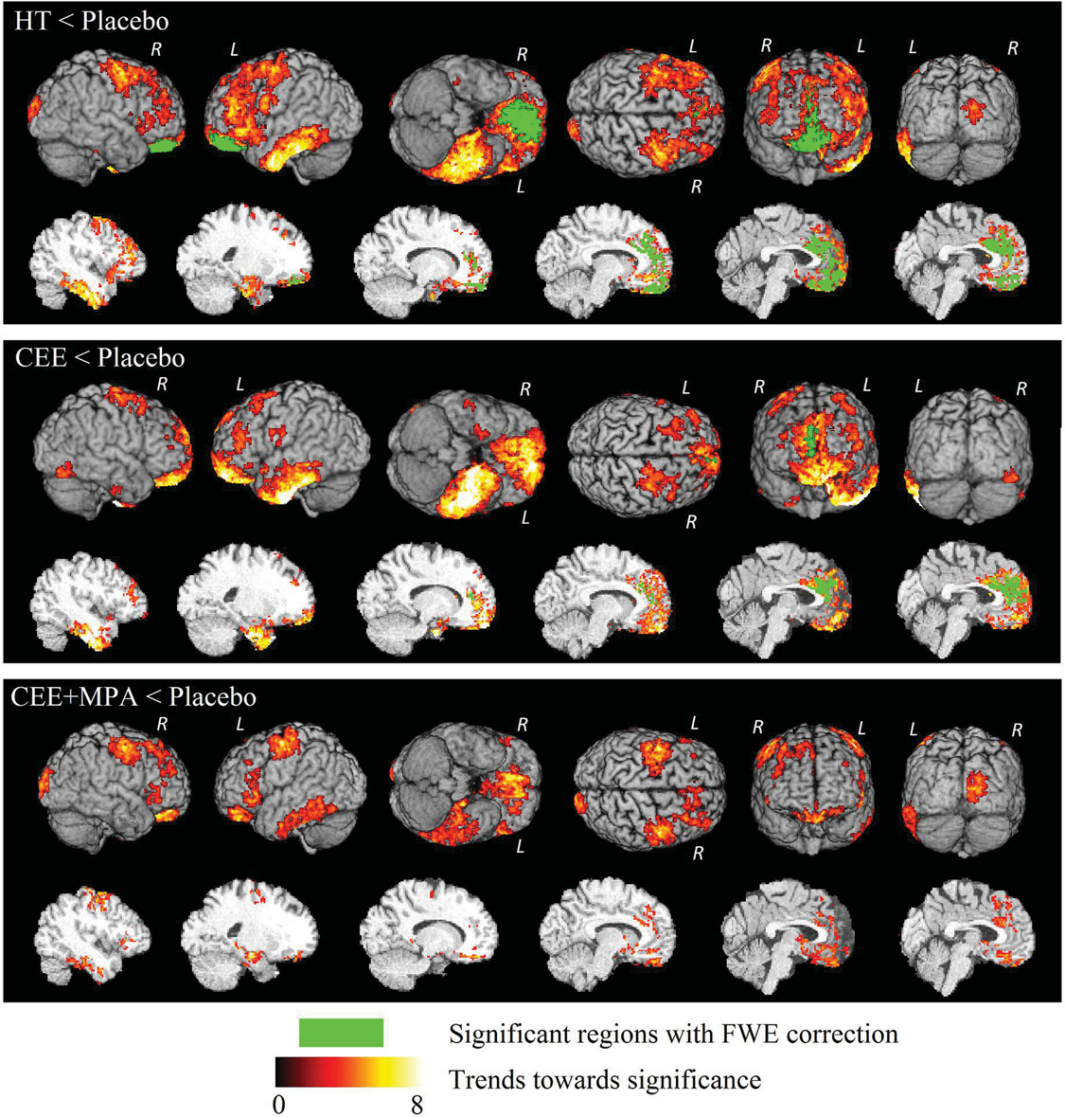
3D surface renderings (upper six images) and representative slices (lower six images) of the ODVBA results on GM volumes comparisons of HT groups < Placebo. Green indicates the detected significant regions with FWE corrected *p* < 0.05; regions of hot colors indicate the trends (uncorrected *p* < 0.05) towards significance characterized by the–log (*p*) values shown in the colorbar.

As shown in Fig 1 and Table 2, women assigned to HT (both CEE-Alone and CEE+MPA users) showed significantly less GM compared to those assigned to placebo in the medial prefrontal regions, including the bilateral anterior cingulate cortex (*p*<0.05, FWE corrected) and the adjacent bilateral superior frontal gyrus (*p*<0.05, FWE corrected) as well as the bilateral middle cingulate cortex (*p*<0.05, FWE corrected), and in the ventromedial prefrontal regions including the orbitofrontal cortex (bilateral: *p*<0.05, FWE corrected) and the gyrus rectus (bilateral: *p*<0.05, FWE corrected).

**Table 2:** The results of GM volume comparisons of HT groups < Placebo. *N* denotes the number of significant voxels in each anatomical region. *t* denotes the *t* value calculated.

Comparisons	Methods	Anatomical Regions	Side	Talairach coordinates			*N*	*t*
				*x*	*y*	*z*		
**HT < Placebo**	FWE(*p* < .05)							
		Anterior Cingulate Cortex	L	-3.96	35.70	14.79	651	4.74
		Medial Superior Frontal Gyrus	L	-3.96	40.13	25.63	449	6.21
		Orbitofrontal Cortex	L	-3.96	49.96	-10.91	448	4.21
		Gyrus Rectus	L	-1.98	39.85	-18.82	333	3.98
		Gyrus Rectus	R	5.94	35.97	-18.62	186	4.89
		Anterior Cingulate Cortex	R	5.94	33.86	16.73	169	4.86
		Orbitofrontal Cortex	R	15.84	37.83	-20.40	95	3.80
		Middle Cingulate Cortex	R	5.94	28.60	28.05	49	2.98
		Middle Cingulate Cortex	L	-3.96	22.88	30.17	25	2.57
		Superior Frontal Gyrus	L	-11.88	28.87	33.56	11	2.84
**CEE-Alone < Placebo**	FWE(*p* < .05)							
		Anterior Cingulate Cortex	L	-3.96	33.77	14.89	491	4.62
		Medial Superior Frontal Gyrus	L	-1.98	43.82	21.76	329	5.64
		Anterior Cingulate Cortex	R	5.94	37.55	12.86	204	5.74
		Medial Superior Frontal Gyrus	R	5.94	50.56	1.15	79	4.12
		Middle Cingulate Cortex	R	7.92	34.41	27.75	14	3.51

The comparison between CEE-Alone treated women and their respective placebo group revealed significantly lower GM volumes in the CEE-Alone group, which were predominantly located in the bilateral anterior cingulate cortex (*p*<0.05, FWE corrected), the bilateral medial part of the superior frontal gyrus (*p*<0.05, FWE corrected).

The CEE+MPA group did not show regions of significantly decreased GM volumes with multiple comparison correction, relative to the matched placebo, but did demonstrate trends toward lower volumes with a liberal uncorrected significance level (S1 Table) in the prefrontal cortex, in accord with the findings in the previous two comparisons. Moreover, all three comparisons revealed trend level volume reductions in left-sided temporal lobe structures (as shown in Fig 1 and S1 Table), including inferior temporal gyrus, parahippocampal gyrus, hippocampus, etc.

#### HT, CEE-Alone, CEE+MPA > Placebo

**Fig 2:**
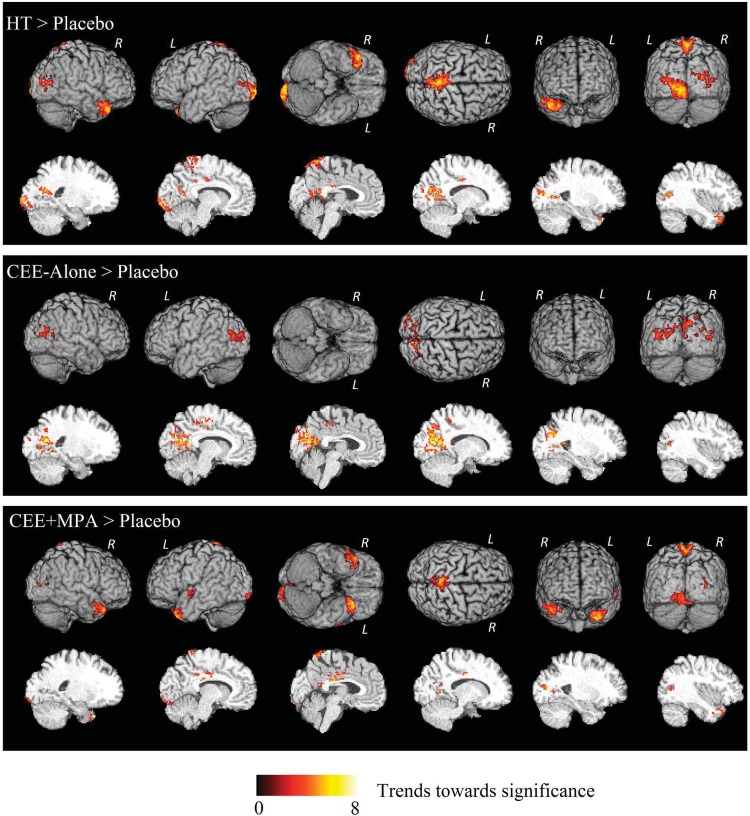
3D surface renderings (upper six images) and representative slices (lower six images) of the ODVBA results on GM volume comparisons of HT groups > Placebo. Regions of hot colors indicate trends (uncorrected *p* < 0.05) towards significance characterized by the–log (*p*) values shown in the colorbar.

There were no significant differences indicative of greater volumes associated with HT surviving the multiple-comparison corrections, but three comparisons showed trends (Fig 2, S1 Table) for the parietal/occipital area, including the right calcarine, the right precuneus, and the left middle occipital gyrus.

### WM Findings

**Fig 3:**
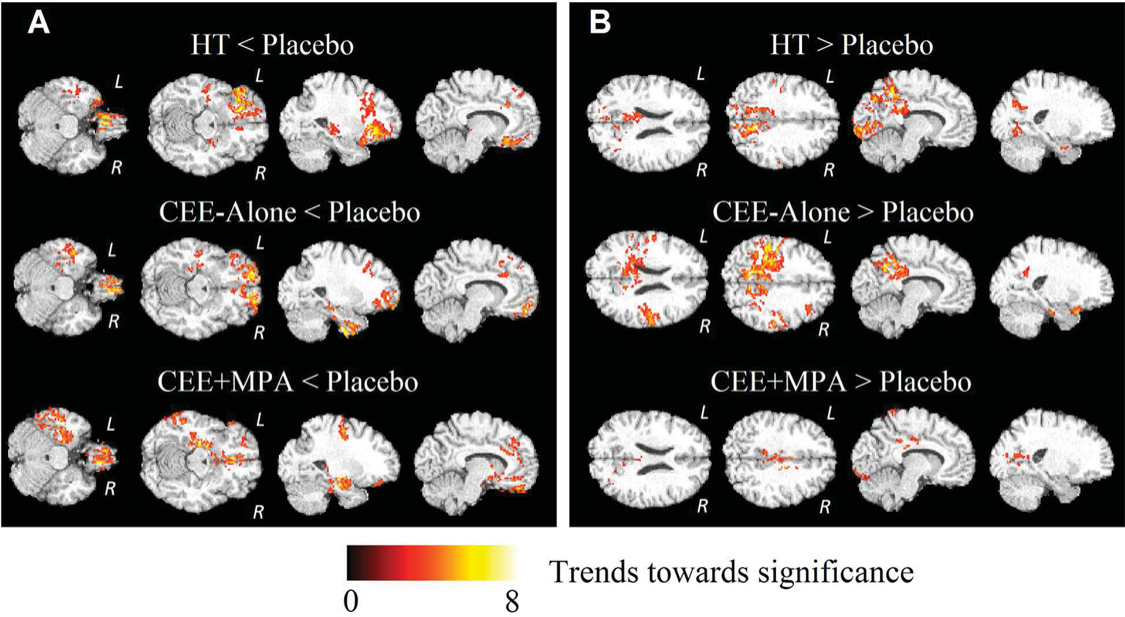
Representative slices of the ODVBA results on WM volume comparisons of (A) HT groups < Placebo, and (B) HT groups > Placebo. Regions of hot colors indicate trends (uncorrected *p* < 0.05) towards significance characterized by the–log (*p*) values shown in the colorbar.

Analysis of local WM volumes revealed no significant differences after multiple comparison correction when comparisons were performed between HT, CEE-Alone, CEE+MPA and the respective placebo groups. However, as demonstrated in Fig 3 and S2 Table, we detected trends that were generally consistent with our findings in GM. Specifically, all three comparisons of HT, CEE-Alone, and CEE+MPA < Placebo showed consistently lower HT-associated WM volumes in regions around the bilateral medial frontal lobe, the bilateral orbitofrontal cortex, and the left temporal lobe. We also found trends toward greater HT-associated WM for all three comparisons for the parietal/occipital area.

### Other Analyses

#### CEE-Alone versus CEE+MPA

**Fig 4:**
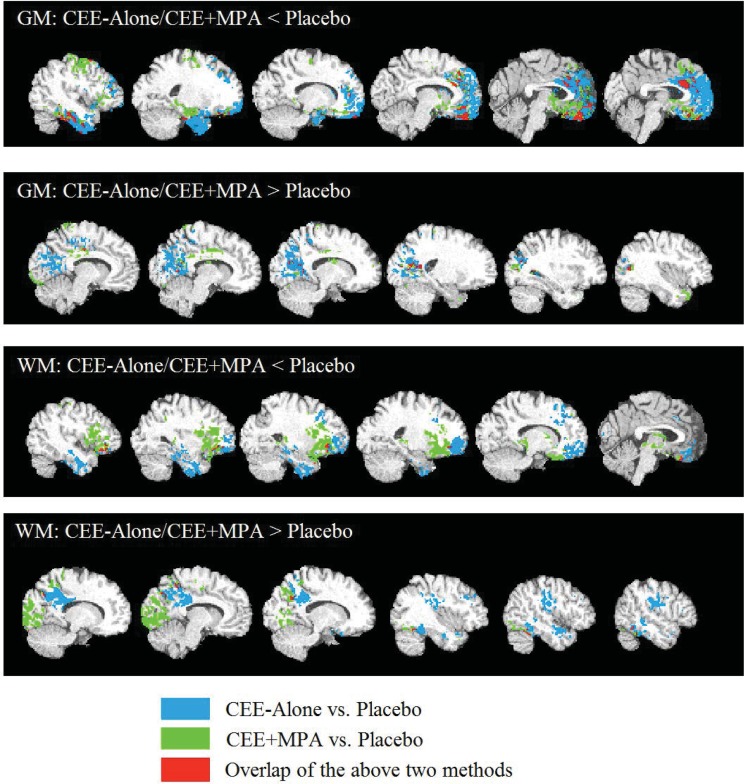
Representative slices of the results of CEE-Alone vs Placebo (light blue) and CEE+MPA vs Placebo (green) on the one standard template. The detected regions are with threshold of uncorrected *p* < 0.05. Red represents the overlap of results between the two methods.

CEE-Alone and CEE+MPA groups cannot be contrasted directly due to differences in their demographic and medical characteristics, requiring separate placebo groups for the two trials. In Fig 4, we demonstrate the overlap of significant findings for GM and WM (regions in red).

#### Sensitivity analysis

As women with other vulnerabilities may be more sensitive to the effects of HT, we recalculated outcomes by excluding women with low baseline cognition (3MS score < 90; 20 CEE subjects and 19 associated placebo; 20 CEE+MPA subjects and 17 associated placebo) and with diabetes (16 CEE subjects and 21 associated placebo; 15 CEE+MPA subjects and 21 associated placebo), which are with small numbers though. This can be regarded as a kind of sensitivity analysis that generally investigates how the uncertainty in the output of a model or system can be distributed to different sources of inputs [38]. The results indicated that the group analyses excluding women with these vulnerabilities yielded the nearly the same patterns with those of the original analyses.

## Discussions

In WHIMS-MRI, we assessed the effects of postmenopausal HT on localized brain volume changes in a large sample of older women in the context of the WHI randomized clinical trials. Women had been previously assigned to either CEE-Alone or CEE+MPA and their respective placebos, allowing investigation of effects of postmenopausal hormone therapy using a novel voxel-based morphometric method to investigate effects across the entire brain. These analyses demonstrated significant regions of HT-associated reductions in brain volume, providing further insights into our understanding of the neurobiological underpinnings of HT effects.

A widespread pattern of significant volume loss was detected in women undergoing HT treatments mainly in the anterior cingulate and the adjacent medial frontal gyrus, and the orbitofrontal cortex. Both anterior cingulate and orbitofrontal cortex are key components of the rostral limbic system [39, 40], which are part of a neural circuit consisting of the anterior component of the limbic system. These structures interface with limbic, executive, and behavioral structures that are involved in the motivational evaluation of stimuli. The two regions and their dense connections are associated with higher-level functions such as decision-making [41, 42, 43] and emotional regulation [44, 45]. The anterior cingulate cortex in particular is involved in the ongoing information processing of cognitive resource allocation [46, 47], that is, “cognitive control” [48]. Impairment of the anterior cingulate cortex and the orbitofrontal cortex has been identified in patients with various types of dementia, including frontotemporal dementia [40, 49], vascular dementia [50, 51] and an agitation subgroup [52] of Alzheimer’s disease (AD).

Temporal lobe structures showing HT-associated adverse effects at the trend level include hippocampus, parahippocampal gyrus, inferior temporal gyrus, and the middle temporal gyrus. The integrity of temporal lobe structures, especially those in the medial aspect of the temporal lobe, are critical to the maintenance of memory function. Medial temporal regions are the earliest to show the neurofibrillary tangle pathology of AD [53, 54] which then spreads to adjacent neocortical areas.

Our current findings are consistent with HT-associated GM reductions demonstrated in our previous WHIMS-MRI studies based on region of interest analysis [5]. In our earlier report, we showed that CEE, with or without MPA, was associated with small but significant decrements in hippocampal and frontal regions. However, our study have provided more accurate localizations of brain volume changes and more refined significance levels across regions, by leveraging on the voxel-wise analysis and multiple comparisons. A follow-up pattern classification analysis [55] selected the most discriminative regions for the classification task and identified associations between CEE-Alone therapy and smaller regional volumes, including inferior temporal gyrus and vicinities of the hippocampus, including the perirhinal cortex and the entorhinal cortex. Moreover, our finding of the adverse effect of HT on brain volume is consistent with a VBM study on a different dataset [11]: in an observational study comparing HT users versus nonusers, Lord and colleagues demonstrated reductions in hippocampal and parahippocampal gyrus volumes in HT users.

In contrast to our findings of multiple regions of HT-associated volume reductions, we found no significant regions where HT was associated with larger volumes compared to placebo. However, at the trend level we found larger volumes in the HT group for the posterior cingulate cortical (PCC) area, including the precuneus and the adjacent calcarine fissure. The PCC/precuneus is a key part of the default mode network [56], which is thought to be important for self-referential [57] and episodic memory [58]. Our findings regarding the PCC area are partially in accordance with the results of the previous studies. An early VBM study comparing HT users versus nonusers [10] identified the posterior cingulate gyrus as a region exhibiting larger volumes among younger estrogen users–women around 50 years old. The pattern classification based study of WHIMS on CEE-Alone versus placebo [55] also identified parietal lobe regions where the CEE group had slightly larger volumes. Further a functional MRI study [59] reported higher posterior cingulate activation in hormone users compared with nonusers during a visual working memory task.

The CEE-Alone and CEE+MPA groups, although compared with different placebo controls, demonstrated similar effects of hormone therapy according to the spatial patterns of structural changes. In Fig 4, we overlaid the results of CEE-Alone and CEE+MPA groups in a single standard template, highlighting regions where the two HT groups share similar structural differences in association with the different interventions. This overlap was most evident in the prefrontal and hippocampal regions, indicating regions of HT-associated GM volume reductions, and in the PCC area which showed higher GM volume in the HT compared with placebo groups. These regions of overlap were consistent with our overall analysis comparing HT vs. placebo, which combined women across both the CEE-Alone and CEE+MPA trials.

Taken together, the most robust findings in our study, which survived more stringent statistical correction, primarily revealed the adverse effect of HT on GM volumes and risk for cognitive impairment and dementia in older women, consistent with prior literature [1, 2, 3, 4, 5, 11, 55, 60, 61, 62]. However, other studies have reported beneficial effects of HT. Two VBM studies [9, 10] indicated higher GM volume in HT users compared with nonusers, which were found widely across the brain gray matter surface. In addition, a few functional neuroimaging studies [59, 63, 64] have also demonstrated that HT can play neuroprotective effects against aging or cognitive decline. However, the majority of studies demonstrating benefits are based on observational studies or studies in younger postmenopausal women. Discrepancies may reflect different experimental strategies and methods used in these studies, as well as differences in the timing of initiation with respect to age and/or the menopausal transition. It should be noted that most studies showing the neuroprotective effects enrolled younger women for their experiments and may reflect a critical window for HT action [65, 66, 67].

Our study involves multiple sites, as most large scale clinical trials do. We promoted standardization across different sites using the ACR phantom QC protocols. The phantom tests have been performed based on a series of measurements, including geometric accuracy, low contrast detectability, high contrast spatial resolution, slice thickness, ghosting artifacts, and more. This phantom test procedure has been a useful method to evaluate the performance of the MRI system, and to determine whether corrective actions are successful. For example, the geometric accuracy test can help to identify gradient mis-calibration and too-low acquisition bandwidth. In addition, after image acquisition, we used the general linear regression model (GLM) as a preprocessing step to further remove the effects of the different clinical sites. Dispersion [68] of different sites can be defined as follows: first, we calculate the sample mean of the whole GM/WM volume values for each site. Next, we calculated the measures of dispersion according to a number of statistics on the sample means, resulting in interquartile range (IQR), mean absolute deviation (MAD), range, and standard deviation (STD). S1 Fig demonstrated the measures of dispersion of different sites, calculated before and after removing the site effects respectively. As shown, the covariance correction has reduced the dispersion of different sites.

Our future work aims to investigate the use of our optimally-discriminative voxel-based morphometric method to study the rate of decline in brain volumes during 4.7 years between the initial and follow-up [69] WHIMS-MRI studies, and also to examine whether the effect of HT on rate of decline differs depending on specific vulnerabilities, e.g. low cognitive function, increased vascular risk, and diabetes.

## Supporting Information

S1 Fig Measures of dispersion of different sites, calculated based on the sample means of the whole A) GM and B) WM volume values.
(DOCX)

S1 Table The results of GM volume comparisons between HT groups and Placebo, obtained with uncorrected *p* value. *N* denotes the number of significant voxels in each anatomical region. *t* denotes the *t* value calculated. (DOCX)

S2 Table The results of WM volume comparisons between HT groups and Placebo, obtained with uncorrected *p* value. *N* denotes the number of significant voxels in each anatomical region. *t* denotes the *t* value calculated. (DOCX)
