# Supplementary material for: Effects of Hormone Therapy on Brain Volumes Changes of Postmenopausal Women Revealed by Optimally-Discriminative Voxel-Based Morphometry
Source: PLoS One. 2016 Mar 14;11(3):e0150834. doi: 10.1371/journal.pone.0150834 (PMC4790922; doi:10.1371/journal.pone.0150834)
Supplement: S1 Fig — Measures of dispersion of different sites, calculated based on the sample means of the whole A) GM and B) WM volume values. (DOCX) [file pone.0150834.s001.docx]

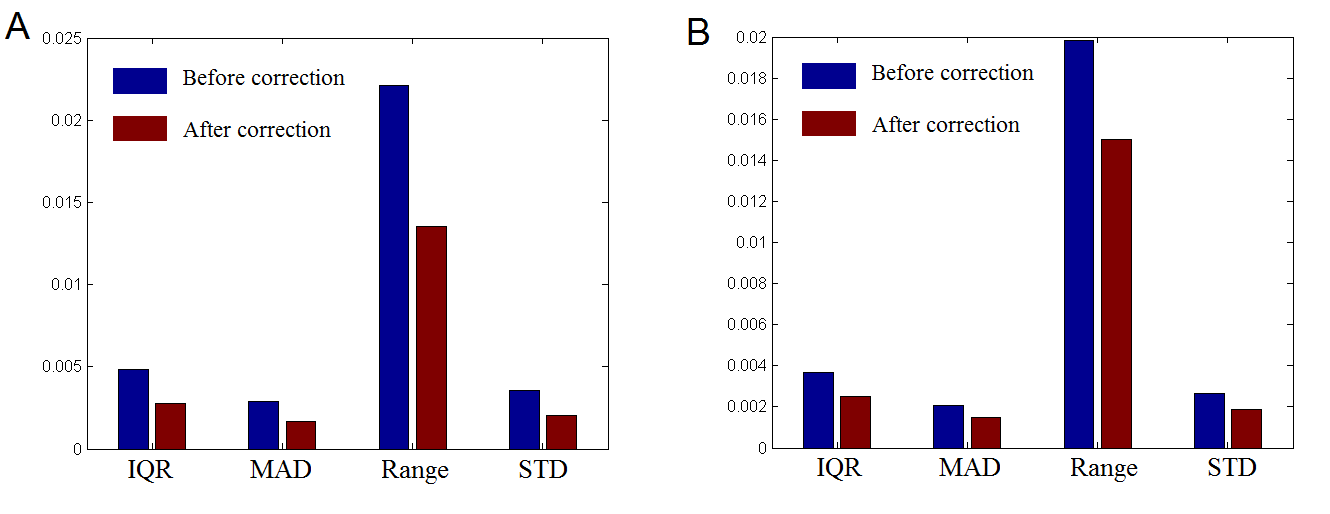


**S1 Figure.** Measures of dispersion of different sites, calculated based on the sample means of the whole A) GM and B) WM volume values.
