# Supplementary material for: Effects of Hormone Therapy on Brain Volumes Changes of Postmenopausal Women Revealed by Optimally-Discriminative Voxel-Based Morphometry
Source: PLoS One. 2016 Mar 14;11(3):e0150834. doi: 10.1371/journal.pone.0150834 (PMC4790922; doi:10.1371/journal.pone.0150834)
Supplement: S1 Table — (DOCX) [file pone.0150834.s002.docx]

**S1 Table**. The results of GM volume comparisons between HT groups and Placebo, obtained with uncorrected *p* value. *N* denotes the number of significant voxels in each anatomical region. *t* denotes the *t* value calculated.

| **Comparisons** | **Methods** | **Anatomical Regions** | **Side** | Talairach coordinates | | | ***N*** | ***t*** |
| --- | --- | --- | --- | --- | --- | --- | --- | --- |
|  |  |  |  | ***x*** | ***y*** | ***z*** |  |  |
| **HT <Placebo** | Uncorrected  (*p*<.001) | Anterior Cingulate Cortex | L | -3.96 | 37.73 | 16.53 | 139 | 6.21 |
|  |  | Medial Superior Frontal Gyrus | L | 0 | 36.43 | 29.50 | 81 | 6.11 |
|  |  | Gyrus Rectus | R | 7.92 | 35.89 | -20.30 | 77 | 4.84 |
|  |  | Orbitofrontal Cortex | L | -9.9 | 43.64 | -20.69 | 71 | 5.2 |
|  |  | Inferior Temporal Gyrus | L | -43.56 | -16.84 | -26.07 | 55 | 6.85 |
|  |  | Anterior Cingulate Cortex | R | 5.94 | 26.20 | 18.95 | 51 | 5.19 |
|  |  | Gyrus Rectus | L | -1.98 | 43.64 | -20.69 | 50 | 4.91 |
|  |  | Orbitofrontal Cortex | R | 17.82 | 37.74 | -22.07 | 14 | 4.00 |
| **CEE-Alone < Placebo** | Uncorrected | Anterior Cingulate Cortex | L | -1.98 | 30.08 | 18.76 | 224 | 5.61 |
|  |  | Inferior Temporal Gyrus | L | -47.52 | -20.71 | -25.88 | 111 | 6.7 |
|  |  | Anterior Cingulate Cortex | R | 5.94 | 31.75 | 13.15 | 86 | 6.02 |
|  |  | Medial Superior Frontal Gyrus | L | 0 | 42.07 | 25.53 | 84 | 5.97 |
|  | (*p*<.001) | Fusiform gyrus | L | -27.72 | -11.20 | -29.72 | 83 | 4.76 |
|  |  | Parahippocampal gyrus | L | -19.8 | -9.18 | -28.14 | 54 | 4.18 |
|  |  | Gyrus Rectus | L | -1.98 | 47.68 | -17.53 | 18 | 5.35 |
|  |  | Medial Superior Frontal Gyrus | R | 3.96 | 47.97 | 27.08 | 16 | 3.69 |
| **CEE+MPA < Placebo** | Uncorrected  (*p*<.001) | Hippocampus | L | -19.8 | -18.19 | -14.23 | 13 | 3.94 |
| **HT > Placebo** | Uncorrected  (*p*<.001) | Calcarine fissure | R | 21.78 | -78.89 | 15.00 | 21 | 5.70 |
| **CEE-Alone > Placebo** | Uncorrected  (*p*<.001) | Calcarine fissure | R | 7.92 | -65.33 | 14.32 | 49 | 5.72 |
|  |  | Precuneus | R | 17.82 | -53.61 | 15.58 | 26 | 5.2 |
|  |  | Cuneus | R | 17.82 | -66.80 | 23.61 | 12 | 4.92 |
|  |  | Middle Occipital Gyrus | L | -27.72 | -74.74 | 20.32 | 11 | 4.00 |
| **CEE+MPA > Placebo** | Uncorrected  (*p*<.001) | \ | \ | \ | \ | \ | \ | \ |
