# Supplementary material for: Effects of Hormone Therapy on Brain Volumes Changes of Postmenopausal Women Revealed by Optimally-Discriminative Voxel-Based Morphometry
Source: PLoS One. 2016 Mar 14;11(3):e0150834. doi: 10.1371/journal.pone.0150834 (PMC4790922; doi:10.1371/journal.pone.0150834)
Supplement: S2 Table — (DOCX) [file pone.0150834.s003.docx]

**S2 Table**. The results of WM volume comparisons between HT groups and Placebo, obtained with uncorrected *p* value. *N* denotes the number of significant voxels in each anatomical region. *t* denotes the *t* value calculated.

| **Comparisons** | **Methods** | **Anatomical Regions** | **Side** | Talairach coordinates | | | ***N*** | ***t*** |
| --- | --- | --- | --- | --- | --- | --- | --- | --- |
|  |  |  |  | ***x*** | ***y*** | ***z*** |  |  |
| **HT <Placebo** | Uncorrected  (*p*<.001) | Orbitofrontal Cortex | L | -29.7 | 28.64 | -9.84 | 31 | 5.79 |
| **CEE-Alone < Placebo** | Uncorrected  (*p*<.001) | Orbitofrontal Cortex | R | 9.9 | 57.71 | -11.30 | 51 | 4.82 |
|  |  | Inferior Temporal Gyrus | L | -47.52 | -9.35 | -31.49 | 47 | 5.45 |
|  |  | Superior Frontal Gyrus | R | 27.72 | 50.84 | 6.67 | 10 | 4.35 |
| **CEE+MPA < Placebo** | Uncorrected  (*p*<.001) | Orbitofrontal Cortex | L | -31.68 | 26.71 | -9.75 | 56 | 4.62 |
|  |  | Insular Cortex | L | -31.68 | 21.23 | -2.74 | 42 | 4.10 |
| **HT >Placebo** | Uncorrected  (*p*<.001) | Precuneus | L | -5.94 | -46.05 | 50.20 | 10 | 4.05 |
| **CEE-Alone > Placebo** | Uncorrected  (*p*<.001) | Precuneus | L | -11.88 | -54.53 | 35.89 | 25 | 4.64 |
|  |  | Inferior Parietal Gyrus | L | -51.48 | -25.29 | 38.11 | 17 | 4.31 |
| **CEE+MPA > Placebo** | Uncorrected  (*p*<.001) | Inferior Temporal Gyrus | L | -33.66 | 6.07 | -33.95 | 28 | 4.45 |
|  |  | Temporal Pole | L | -31.68 | 7.84 | -37.40 | 14 | 4.60 |
